# A pilot trial of consolidation bevacizumab after hypo‐fractionated concurrent chemoradiotherapy in patients with unresectable locally advanced non‐squamous non‐small‐cell lung cancer

**DOI:** 10.1002/cam4.6381

**Published:** 2023-08-03

**Authors:** LanQing Huo, Chu Chu, XiaoBo Jiang, ShiYang Zheng, PengXin Zhang, Rui Zhou, NaiBin Chen, JinYu Guo, Bo Qiu, Hui Liu

**Affiliations:** ^1^ Department of Radiation Oncology Sun Yat‐sen University Cancer Center Guangzhou China; ^2^ State Key Laboratory of Oncology in South China Sun Yat‐sen University Cancer Center Guangzhou China; ^3^ Collaborative Innovation Center for Cancer Medicine Sun Yat‐sen University Cancer Center Guangzhou China; ^4^ Lung Cancer Institute of Sun Yat‐sen University Guangzhou China; ^5^ Guangdong Association Study of Thoracic Oncology Guangzhou China

**Keywords:** consolidation bevacizumab, hypo‐CCRT, LA‐NS‐NSCLC, treatment‐related toxicity

## Abstract

**Purpose:**

To determine the feasibility of incorporating bevacizumab consolidation into hypo‐fractionated concurrent chemoradiotherapy (hypo‐CCRT) for patients with unresectable locally advanced non‐squamous non‐small‐cell lung cancer (LA‐NS‐NSCLC).

**Patients and Methods:**

Eligible patients were treated with hypo‐RT (40Gy in 10 fractions) followed by hypo‐boost (24‐28Gy in 6–7 fractions), along with concurrent weekly chemotherapy. Patients who completed the hypo‐CCRT without experiencing ≥G2 toxicities received consolidation bevacizumab every 3 weeks for up to 1 year, until disease progression or unacceptable treatment‐related toxicities.

The primary endpoint was the risk of G4 or higher hemorrhage. Secondary endpoints included progression‐free survival (PFS), overall survival (OS), locoregional failure‐free survival (LRFS), distant metastasis‐free survival (DMFS), and objective response rate (ORR). All time‐to‐event endpoints (OS, PFS, LRFS, and DMFS) were measured from the start of radiotherapy.

**Results:**

Between December 2017 and July 2020, a total of 27 patients were included in the analysis, with a median follow‐up duration of 28.0 months. One patient (3.7%) developed G5 hemorrhage during bevacizumab consolidation. Additionally, seven patients (25.9%) had G3 cough and three patients (11.1%) experienced G3 pneumonitis. The ORR for the entire cohort was 92.6%. The median OS was 37.0 months (95% confidence interval, 8.9–65.1 months), the median PFS was 16.0 months (95% confidence interval, 14.0–18.0 months), the median LRFS was not reached, and the median DMFS was 18.0 months.

**Conclusions:**

This pilot study met its goal of demonstrating the tolerability of consolidation bevacizumab after hypo‐CCRT. Further investigation of antiangiogenic and immunotherapy combinations in LA‐NSCLC is warranted, while the potential for grade 3 respiratory toxicities should be taken into consideration.

## Graphical abstract

It demonstrated that acceptable safety of consolidation therapy with bevacizumab after hypo‐CCRT in selected patients with LA‐NSCLC and that consolidation therapy with bevacizumab resulted in encouraging outcomes, while toxicity is of particular concern. Respiratory toxicity had been reported as the leading cause of fatal adverse event among patients receiving CCRT and consolidation immunotherapy.

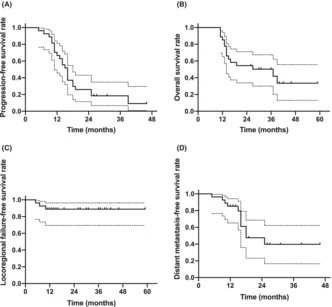

## 1 INTRODUCTION

Lung cancer is the leading cause of cancer‐related deaths worldwide.^1^ Non‐small‐cell lung cancer (NSCLC) accounts for 80%–85% of cases, with stage III representing 30% of those cases.^2^ Concurrent chemoradiotherapy (CCRT) has been adopted as the recommended treatment for individuals with unresectable or inoperable stage IIIA and IIIB NSCLC with satisfactory performance status supported by many phase III studies.^3^ Earlier phase I/II trials revealed a modestly favorable correlation between increased the biologically effective dose (BED) and improved survival outcomes.^4^ Based on the findings of the RTOG 0617 trial^5^ and the encouraging results of SBRT in treating medically inoperable early‐stage lung cancer,^6^ fraction dose modification may be a preferable option to total dose escalation.^4^ Several phase I dose escalation studies have demonstrated that thoracic hypo‐fractionated radiotherapy, with a total dose of up to 60Gy at 3–5Gy per fraction, was generally well‐tolerated.^7^ In a clinical trial conducted by Kong, radical‐intent hypo‐fractionated radiotherapy with a total dose of 60Gy delivered in 15 fractions showed satisfactory survival outcomes in locally advanced non‐squamous non‐small‐cell lung cancer (LA‐NS‐NSCLC) patients, with a 47‐month median overall survival (OS) and median progression‐free survival (PFS) of 16 months.^8^

Despite the improved efficacy achieved with hypo‐fractionated radiotherapy, distant metastases remained the common failure patterns during long‐term follow‐up. Immune checkpoint inhibitors have revolutionized standard treatment and survival expectations for NSCLC patients. The PACIFIC study demonstrated that durvalumab, an immune checkpoint inhibitor, provided considerably increased PFS and OS than placebo in stage III unresectable NSCLC patients who did not progress following concurrent chemoradiotherapy (median PFS 16.8 months vs. 5.6 months; median OS 23.2 months vs. 14.6 months; *p* < 0.001).^9^ These practice‐changing results have sparked enthusiasm for further research into immunotherapy and combination treatments in this setting.

Bevacizumab, when combined with carboplatin and paclitaxel, has shown improved response rates and delayed disease progression in chemo‐naive patients with advanced NSCLC in a randomized Phase III trial.^10^ The addition of bevacizumab to consolidation therapy improves response rates, PFS, and OS in LA‐NS‐NSCLC. Therefore, there has been a surge of interest in studying this therapy in patients with earlier stages of the disease.^11^, ^12^ In the pilot trial SWOG S0533, the median PFS was 38 months (95% CI: 23–46 months) in low‐risk NSCLC patients. Furthermore, besides its established antiangiogenic properties, suppression of vascular endothelial growth factor (VEGF) has several immunomodulatory effects and may enhance the effectiveness of immunotherapy.^13^, ^14^ There are ongoing trials evaluating the combination of bevacizumab and immunotherapy in NSCLC. A systematic review enrolling 54 randomized controlled trials suggests adding bevacizumab to chemotherapy‐immunotherapy may provide additional therapeutic benefits without increasing treatment burden.^15^

The aim of this trial was to determine the feasibility of incorporating consolidation bevacizumab into hypo‐fractionated CCRT for unresectable LA‐NS‐NSCLC and to provide information for further investigating the combination of bevacizumab with consolidation immunotherapy.

## 2 METHODS

### 2.1 Eligibility and exclusion criteria

Patients were recruited between June 2018 and June 2020. Patient eligibility criteria included (1) age between 18 and 75; (2) histologically confirmed non‐squamous NSCLC; (3) unresectable stage III disease based on the 8th edition of the TNM staging system recommended by the American Joint Committee on Cancer (AJCC); (4) measurable lesions per Response Evaluation Criteria in Solid Tumors (RECIST) criteria; (5) Eastern Cooperative Oncology Group (ECOG) performance status score of 0–1; (6) Charlson Comorbidity Index score ≤4; (7) Functions of the bone marrow, liver, and kidneys are adequate. The main exclusion criteria included (1) contraindication for radiotherapy or chemotherapy; (2) histologically confirmed squamous cell carcinoma; (3) pathologic condition with high bleeding risk, ulcers, non‐healing wounds, or fractures of the bones; (4) prior malignancies, except for cervical in situ or curable non‐melanoma skin cancer; (5) distant metastases.

### 2.2 Ethics

The institutional review board granted regulatory approval, and the study was carried out in conformity with the Declaration of Helsinki. Before obtaining any trial‐specific procedures, written informed consent was provided by all participants.

### 2.3 Hypo‐fractionated radiotherapy and hypo‐fractionated boost with concurrent weekly chemotherapy

**FIGURE 1:**
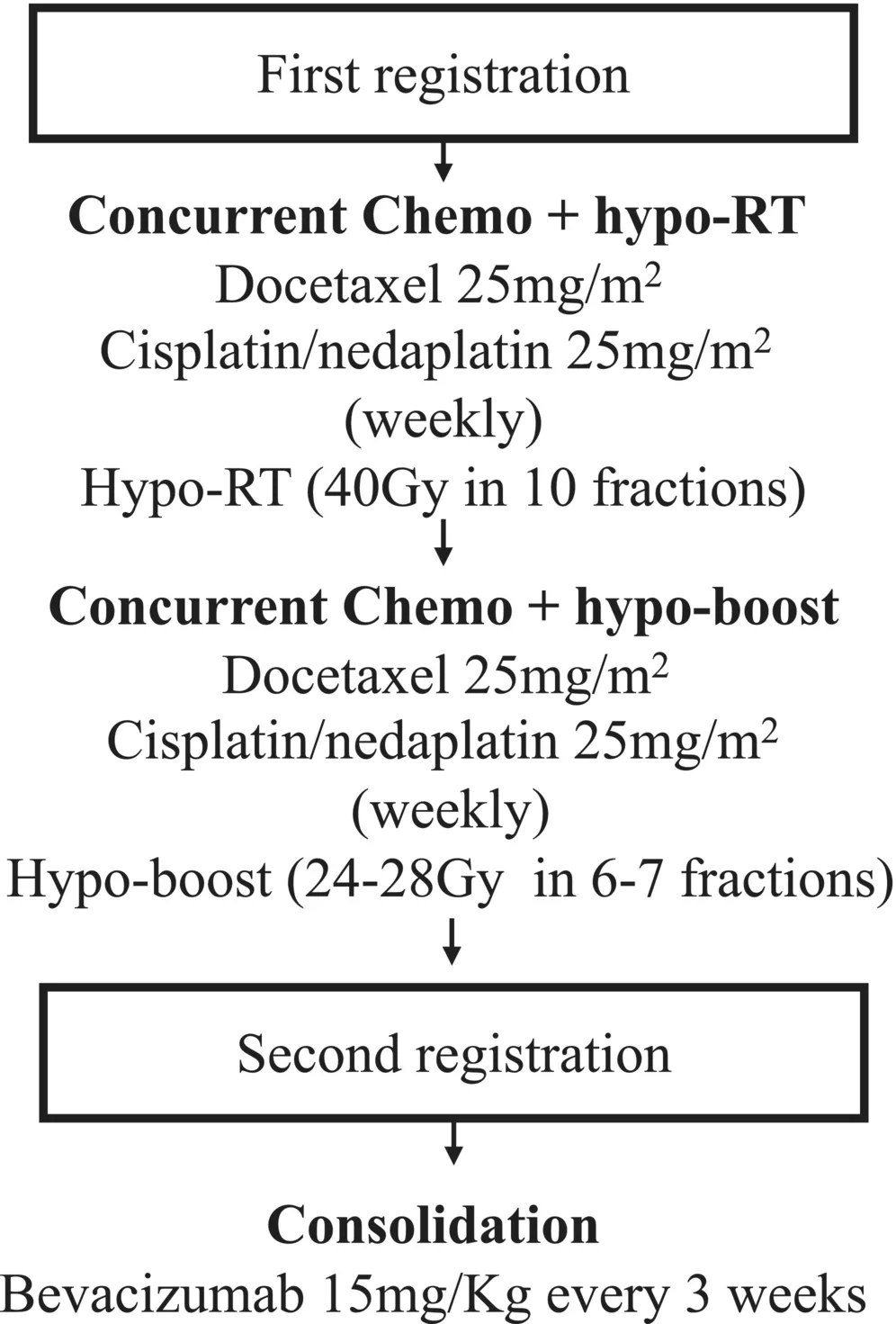
Treatment schema of study.

A four‐dimensional CT (4D‐CT) scan was performed on patients who were immobilized on a vacuumed pad in the supine position with both arms straight beside the torso. The visible main tumor and metastatic lymph nodes detected by pre‐treatment positron emission tomography‐computed tomography (PET‐CT) or regular contrast‐enhanced CT scans were included in the gross tumor volumes (GTVs). The GTVs were manually contoured on the 4D‐CT using the reconstructed maximum intensity projection data set of the 10 respiratory phases. The planning target volumes (PTVs) encompassed the GTV with a 5 mm isotropic margin. Thoracic radiotherapy was delivered using the IMRT approach, with daily cone‐beam CT imaging guidance to confirm tumor localization and assure treatment accuracy. The dose of 40Gy with a fraction dose of 4Gy was delivered to PTV‐GTV as the hypo‐RT course. Following that, a re‐evaluation comprising of a chest/upper abdominal contrast‐enhanced CT scan and a pulmonary function test was performed after a 2‐week break to assess residual disease and patients' physical status. Patients were eligible for receiving a hypo‐boost therapy when they had no disease progression and no persistent ≥ Grade 2 treatment‐related toxicities. Every 2 weeks, patients who had persistent grade 2/3/4/5 toxicities were to be reevaluated to see whether they were still eligible for the hypo‐boost. Eligible patients underwent a second CT simulation and four‐dimensional CT scan to reformulate the adaptive radiotherapy plan. The adaptive plan of a dose 24‐28Gy in 6–7 fractions (4Gy per fraction) was delivered to the residual tumor (PTV‐GTV‐residual) as the hypo‐boost **(**Figure 1). It was required that at least 95% of the PTV volume got 95% of the prescribed dosage. Dose constraints were provided as Table S1.

Docetaxel (25 mg/m^2^) plus cisplatin/nedaplatin (25 mg/m^2^) were infused into the patients per week concurrently with hypo‐RT and hypo‐boost therapy. For the course of the treatment, four rounds of chemotherapy were intended. Given the potential for adverse reactions, the dosage of chemotherapy could be adjusted at clinical discretion.

### 2.4 Consolidation Bevacizumab after hypo‐CCRT

Patients were eligible for receiving the consolidation bevacizumab when they had no disease progression and no persistent ≥ G2 toxicities within 2 months after CCRT. Consolidation therapy of bevacizumab with a dose of 15 mg/kg on Day 1 of every 21‐day cycle was commenced within 1–2 months after CCRT for up to 1 year, or disease progression or unacceptable treatment‐related toxicities. The criteria for the discontinuation of consolidation treatment included any ≥Grade 3 non‐hematological toxicities or ≥ grade 2 hemoptysis, or disease progression.

### 2.5 Toxicity and adverse events

All therapy‐related adverse events were accurately documented during weekly visits and following treatment review. Acute toxicities were examined from the start of hypo‐CCRT to 90 days after the end of therapy or the commence of new treatment, and late toxicities were recorded thereafter till 1 year after the end of therapy or the commence of new treatment. All treatment‐related toxicity was rated using the National Cancer Institute's Common Terminology Criteria for Adverse Events (CTCAE) (version 5.0).

### 2.6 Response evaluation and follow‐up

The treatment response to hypo‐CCRT was evaluated 6–8 weeks after the completion of hypo‐CCRT using a series of clinical work‐ups including patients' history, physical examination, blood routine, serum chemistries, heart echocardiography, pulmonary function test (PFT), contrast‐enhanced brain magnetic resonance imaging (MRI), and chest and upper abdominal contrast‐enhanced CT. PET‐CT scans were recommended. As per the RECIST criteria, treatment responses can be classified as complete responses (CR), partial responses (PR), stable diseases (SD), and progressive diseases (PD). Every 3 months for the first 2 years, every 6 months for years three through five, and once a year after that, chest and abdomen CTs were taken. Over the first 2 years, every 6 months, and subsequently annually, brain MRI scans were conducted. A PET‐CT scan or a bone scan was performed when the patient's presence of distant metastases was thought to be probable.

### 2.7 Statistical analysis

The primary objective was to assess the risk of grade 4 or higher hemorrhage associated with the addition of bevacizumab to hypo‐CCRT. A one‐sided test based on a binomial distribution with a significance level of 5% and 27 patients enrolled in consolidation therapy were sufficient to distinguish between the null hypothesis of a 20% rate of grade 4 or higher hemorrhage and the alternative hypothesis of an acceptable rate (5%) of these toxicities with 80% exact power.

Second endpoints included progression‐free survival (PFS), which was measured from the initiation of radiation until the first sign of disease progression or death. Overall survival (OS), locoregional failure‐free survival (LRFS), distant metastasis‐free survival (DMFS), objective response rate (ORR), and toxicity were the secondary objectives. All time‐to‐event endpoints (OS, PFS, LRFS, and DMFS) were calculated starting from the beginning of radiation. The ORR was calculated as the sum of the CR and PR rates.

Descriptive statistics were used to analyze baseline characteristics. The Wilcoxon test was used to compare median values, whereas the chi‐square test was used to compare proportions. The Kaplan–Meier method was used to calculate survival rates, and the long‐rank test was performed to look for variations in survival curves. SPSS software, version 22.0, was used for all data analyses. Statistical significance was defined as a *p*‐value of 0.05.

## 3 RESULTS

### 3.1 Accrual and disposition of patients

**FIGURE 2:**
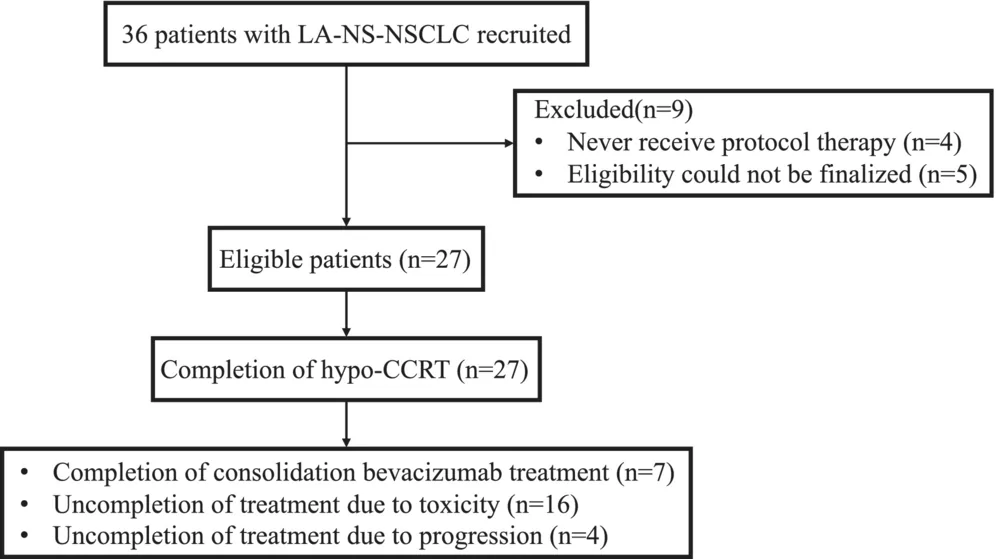
Flow diagram of all patients (*n* = 27).

A total of 36 patients were enrolled in the study between December 1, 2017, and July 31, 2020. Out of the 36 patients, 31 patients were deemed eligible, while five were ineligible due to reasons such as distant metastases or contraindication for radiotherapy or chemotherapy. Four patients did not receive protocol‐specific therapy. A total of 27 patients completed hypo‐CCRT and were subsequently registered to consolidation therapy (Figure 2). The demographics and disease characteristics of these 27 patients are summarized in Table 1. The median age of the patients was 57 years, with 22 males and five females. All 27 patients have an ECOG performance status score of zero. Three patients had epidermal growth factor receptor (EGFR) mutation status, while 22 were wild type. All patients' histology was adenocarcinoma. Smokers constituted 88.9% of all patients. Prior to hypo‐CCRT, 14 patients had received induction chemotherapy.

**TABLE 1:** Baseline patient characteristics (*n* = 27).

Characteristic	No. (%)
Age (years)	
Median	57
Range	41–71
Gender	
Male	22 (81.5)
Female	5 (18.5)
ECOG PS	
0	17 (62.9)
1	10 (37.1)
EGFR mutation status	
Mutated	3 (11.2)
Wild type	22 (81.4)
Unknown	2 (7.4)
Histology	
Adenocarcinoma	27 (100.0)
Disease stage	
IIIA	12 (44.5)
IIIB	9 (33.3)
IIIC	6 (22.2)
Location	
Left lung	10 (37.0)
Right lung	17 (63.0)
Smoking history	
Yes	24 (88.9)
No	3 (11.1)
Induction chemotherapy before hypo‐CCRT	
Yes	14 (51.9)
No	13 (48.1)

### 3.2 Treatment compliance

All of the 27 patients completed hypo‐CCRT (100%). The dosimetry information is shown in Table S2. Bevacizumab was delivered with a median of 7 cycles (range 1–32). Seven of the 27 patients completed the full course of bevacizumab consolidation (25.9%). In 59.3% of cases, toxicities were cited as the main reason for discontinuing consolidation treatment, followed by disease progression in 14.8% of cases.

### 3.3 Toxicity

Data in Table 2 show toxicities that occurred during or after the consolidation therapy. Grade 4 or higher hemorrhage was noted in one patient (3.7%) manifesting as fatal hemoptysis. Other ≥Grade 3 toxicities included cough, hemoptysis, pneumonitis, enteritis, lymphopenia, hypertension, and pain. The most prominent ≥Grade 3 toxicity was cough (25.9%), followed by ≥Grade 3 lymphopenia (14.8%), pain (14.8%), and pneumonitis (11.1%).

**TABLE 2:** Toxicities related to treatment protocol for the whole cohort (n = 27).

Toxicity	Grade, *n* (%)					
	0	1	2	3	4	5
Pulmonary						
Cough	4 (14.8)	7 (25.9)	9 (33.3)	7 (25.9)	0	0
Pulmonary fibrosis	21 (77.8)	6 (22.2)	0	0	0	0
Hemoptysis	23 (85.2)	3 (11.1)	0	0	0	1 (3.7)
Pneumonitis	1 (3.7)	8 (29.6)	15 (55.5)	3 (11.1)	0	0
Gastrointestinal						
Esophagitis	18 (66.6)	4 (14.8)	5 (18.5)	0	0	0
Enteritis	23 (85.1)	2 (7.4)	1 (3.7)	1 (3.7)	0	0
Hematologic						
Anemia	22 (81.5)	5 (18.5)	0	0	0	0
Leukopenia	25 (92.6)	2 (7.4)	0	0	0	0
Lymphopenia	7 (25.9)	6 (22.2)	10 (37.1)	4 (14.8)	0	0
Neutropenia	26 (96.3)	1 (3.7)	0	0	0	0
Thrombocytopenia	25 (92.6)	2 (7.4)	0	0	0	0
Elevated creatine	23 (85.2)	4 (14.8)	0	0	0	0
General						
Hypertension	7 (25.9)	11 (40.7)	7 (25.9)	2 (7.4)	0	0
Arrhythmia	24 (88.9)	1 (3.7)	2 (7.4)	0	0	0
Dermatitis	25 (92.6)	2 (7.4)	0	0	0	0
Pain	10 (31.1)	7 (25.9)	6 (22.2)	4 (14.8)	–	–

### 3.4 Treatment response and survival

The best overall response and survival was reported for the 27 patients who registered for the consolidation therapy. Three (11.1%), 22 (81.5%), and 2 (7.4%) out of 27 patients had confirmed CR, PR, and SD, respectively. The ORR for the entire cohort was 92.6% (Table 3).

**TABLE 3:** Treatment details and responses of all patients (*n* = 27).

Treatment compliance, *n* (%)	
Completion of treatment	7/27 (25.9)
Reason of uncompletion of treatment, *n* (%)	
Toxicity	16/27 (59.3)
Progression	4/27 (14.8)
RT dose received (cGy)	
Median	6400
Range	4000 ~ 7500
Compliance of weekly concurrent chemotherapy, *n* (%)	
1 cycle	2/27 (7.4)
2 cycles	19/27 (70.3)
3 cycles	5/27 (18.5)
4 cycles	1/27 (3.7)
Bevacizumab cycle	
Median	7
Range	1 ~ 32
Clinical response after radiotherapy, *n* (%)	
CR	3/27 (11.2)
PR	22/27 (81.4)
SD	2/27 (7.4)

**FIGURE 3:**
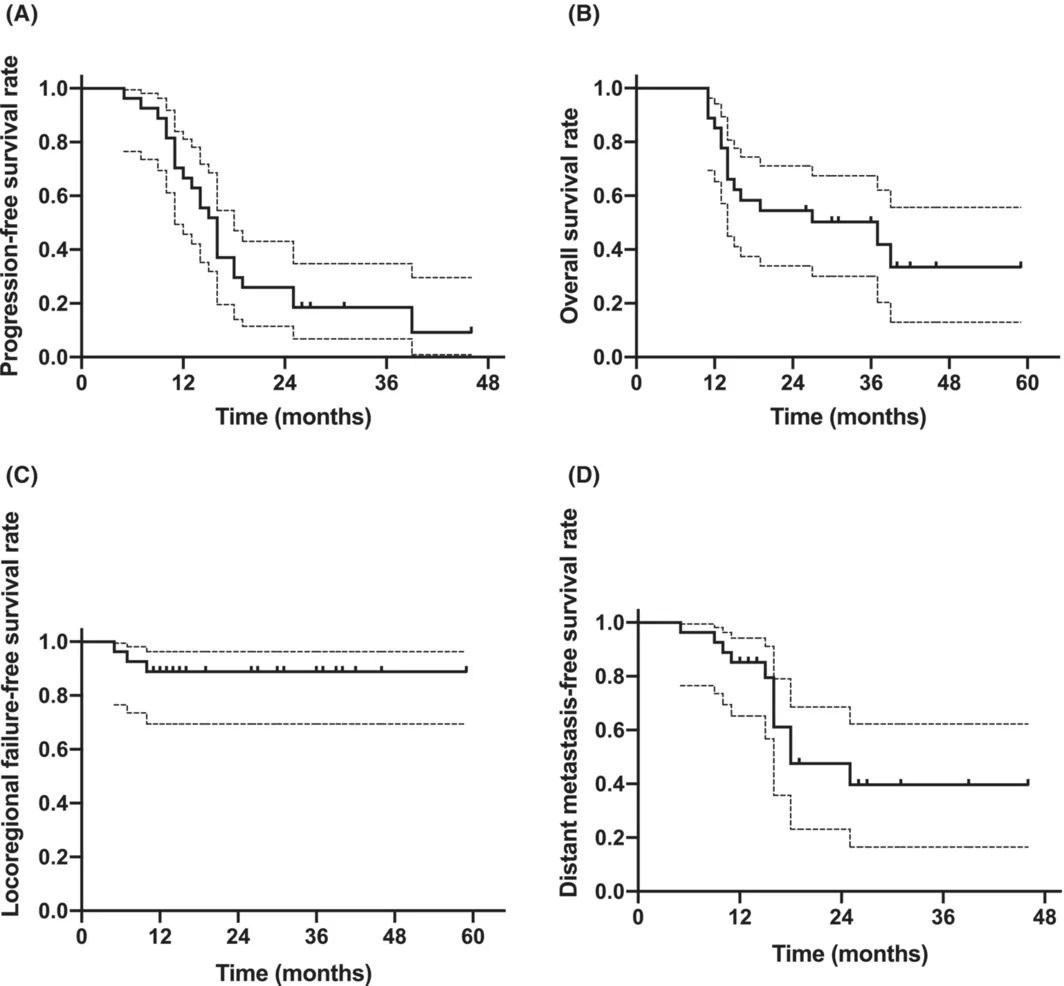
Clinical outcomes (A) OS, (B) PFS, (C) LRFS, and (D) DMFS in all patients (*n* = 27). DMFS, Distant metastasis‐free survival; LRFS, locoregional failure‐free survival; OS, overall survival; PFS, progression‐free survival.

It was found that the median OS was 37.0 months (95% CI, 8.9–65.1 months). The one‐year OS was 85.2%, and two‐year OS was 54.4%. The median PFS was 16.0 months (95% CI, 14.0–18.0 months). The 1‐year PFS was 66.7%, and 2‐year PFS was 25.9%. The median LPFS was not met, and the median DMFS was 18.0 months (95% CI, 8.0–28.0 months). The 1‐year LPFS was 77.8%, and two‐year LPFS was 51.9%. The one‐year DMFS was 85.2%, and two‐year DMFS was 47.6% (Figure 3).

### 3.5 Failure patterns and salvage treatment

At the last follow‐up, 13 patients had disease progression. Among these patients, two of them had local recurrence and eleven of them had distant metastasis. Metastasis most commonly occurred in the brain. Following disease progression, six patients were treated with radiotherapy, including SBRT and X knife. Another three patients received tyrosine kinase inhibitors. Another two patients were treated with chemotherapy.

## 4 DISCUSSION

This pilot research demonstrated acceptable safety of bevacizumab consolidation therapy following hypo‐CCRT in carefully selected group of patients with LA‐NSCLC, meeting its primary endpoint with 3.7% of patients experiencing a G4 hemorrhage. Additionally, consolidation therapy with bevacizumab yielded encouraging outcomes. The ORR for the entire cohort was 92.6%. The median OS was 37.0 months (95% CI, 8.9–65.1 months). The 1‐year OS was 85.2%, and 2‐year OS was 54.4%. The median PFS was 16.0 months (95% CI, 14.0–18.0 months). The 1‐year PFS was 66.7%, and 2‐year PFS was 25.9%.

Bevacizumab has shown improved outcomes in various cancer types, including non‐squamous NSCLC, cervical cancer, ovarian cancer, colorectal cancer, renal cell carcinoma, and glioblastoma, when combined with chemotherapy.^16^ However, bevacizumab is closely related to a variety of toxicities, such as hypertension, vascular events, delayed wound healing, proteinuria, and tracheoesophageal fistula. Although adding bevacizumab into chemotherapy was well tolerated as shown by PointBreak trial,^17^ a study investigating the feasibility of incorporating bevacizumab into CCRT for locally advanced NSCLC was prematurely closed due to severe massive hemoptysis.^18^ Tracheoesophageal fistula was found to be a common complication following concurrent thoracic chemoradiation and bevacizumab.^18^, ^19^, ^20^ Two independent phase II clinical trials combining chemotherapy and radiation with bevacizumab were conducted in small‐cell lung cancer and NSCLC. Preliminary efficacy and safety results from these two studies indicated an increased incidence of tracheoesophageal fistulae in either small‐cell lung cancer or NSCLC settings.^19^ All patients who developed tracheoesophageal fistulae had severe esophageal toxicity after CCRT and concurrent bevacizumab treatment.^19^ This suggested that severe esophagitis after CCRT is a risk factor for subsequent esophageal fistula. In Socinski et al.'s study on locally advanced NSCLC,^20^ patients received induction carboplatin/paclitaxel/bevacizumab, weekly carboplatin/paclitaxel and bevacizumab (every other week), erlotinib with 74 Gy thoracic conformal radiotherapy, and consolidation bevacizumab and erlotinib. The trial was terminated for patients with squamous cell histology due to two grade 5 hemorrhagic episodes occurring after CCRT, 78 and 69 days after the last dose of bevacizumab, respectively. In summary, current evidence does not support the use of bevacizumab in concurrent settings with CCRT in locally advanced NSCLC. The mucosal injury, impaired neovascularization, and healing caused by combined modality treatment may contribute to these fatal toxicities.

Based on the above toxicity reports, in order to reduce the occurrence of severe side effects such as massive hemorrhage and esophageal fistula, the current study used bevacizumab only in consolidation phase. Bevacizumab was administered after all radiation toxicity had resolved, including esophagitis, and patients had fully recovered. In this trial, consolidation bevacizumab demonstrated a tolerable toxicity profile when combined with hypo‐CCRT. However, it is important to note that there were seven patients (25.9%, 7/27) experienced G3 persistent cough, three patients (11.1%, 3/27) suffered G3 pneumonitis during consolidation, another four patients (14.8%, 4/27) experienced chest discomfort without disease progression, which greatly impeded normal activities and could not be relieved by morphine. On RTOG 0617, the rates of grade 2 pneumonitis were 4% versus 8% (60 vs. 74 Gy), grade 3 pneumonitis 4% versus 0%, grade 3 cough 3% versus 5%. Although the current trial achieved its primary endpoint, it reported a higher pulmonary toxicity rate compared with RTOG0617. These treatment‐related toxicities should be taken into consideration when designing future studies that combine consolidation immunotherapy with bevacizumab.

The safety profile of hypo‐CCRT in LA‐NSCLC, particularly in the context of consolidation therapy, is an important consideration. In the RTOG 1106 study, LA‐NSCLC patients underwent CCRT with a total dose of 40–50 Gy, followed by a mid‐treatment PET‐CT scan within 2 weeks. Adaptive radiation therapy was then delivered to residual disease.^21^ The median OS was 25 months, and the 2‐year OS rate was 52%. The incidence of G3 radiation‐induced esophagitis and pneumonitis was 12% and 7%, respectively. In our previous study, we analyzed 75 LA‐NSCLC patients who were treated with hypo‐RT (4 Gy per fraction) and hypo‐boost combined with weekly concurrent chemotherapy.^22^ The median PFS was 21.6 months, and the median OS had not been reached. G3 acute radiation esophagitis was observed in 5.3% of patients. About 17.3% of patients experienced G2 pneumonitis, and no G3‐5 acute pneumonitis occurred during follow‐up. These findings suggest that the use of hypo‐RT (4 Gy per fraction) and hypo‐boost may help mitigate treatment‐related toxicities and provide an opportunity for further consolidation therapy.

In addition to its antiangiogenic effects,^23^, ^24^ the inhibition of vascular endothelial growth factor (VEGF) can also modulate immunity as well.^25^, ^26^ By reversing VEGF‐mediated immunosuppression, bevacizumab has the potential to enhance the effectiveness of immunotherapy.^27^, ^28^, ^29^ Combining immunotherapy with antiangiogenesis treatment may alter the hostile tumor microenvironment (TME) from an immune‐suppressive one to an immune‐active one.^30^, ^31^ This change in the TME can promote tissue perfusion and enhance immune cell infiltration, thus augmenting the impact of immunotherapy.^32^ There is evidence to support this notion, as the activation and reprogramming of immune system cells can in turn promote the normalization of tumor vascular system. In the IMpower150 clinical trial, improved survival was observed in chemotherapy‐naive metastatic NSCLC patients treated with atezolizumab plus bevacizumab plus carboplatin plus paclitaxel compared with those who received bevacizumab plus carboplatin plus paclitaxel in the intention‐to‐treat population, and in patients with baseline liver metastases.^33^ Based on the above evidence, as well as the results of PACIFIC trial, combining immunotherapy and bevacizumab in consolidation setting provided an option to further improve the outcome. However, the potential for toxicity is of particular concern. Respiratory toxicity has been reported as the leading cause of fatal adverse event among patients receiving CCRT and consolidation immunotherapy. The current study found 25.9% of patients experienced G3 persistent cough, highlighting the importance of monitoring and managing respiratory tract toxicity associated with consolidative treatment.

The current study had several limitations. Firstly, it was mostly conducted on the indigenous population in a single institution. Treatment outcomes might fluctuate based on socioeconomic position and demographic variables when the strategy was applied to diverse patient populations. Secondly, the median overall survival was 37 months, which was better than earlier studies and might be due to the stringent eligibility criteria and limited number of patients included. The single arm study design limits the direct comparison to conventionally fractionated regimens. Lastly, we commenced this study since 2017 when consolidation immunotherapy was not part of the standard practice. Combination of immunotherapy with anti‐angiogenesis treatment in consolidation setting warrants further investigation. The slightly higher respiratory toxicity should be considered in combination therapy.

## 5 CONCLUSION

This pilot study met its goal of demonstrating the tolerability of consolidation bevacizumab after hypo‐CCRT. Further investigation of antiangiogenic and immunotherapy combinations in LA‐NSCLC is warranted while G3 respiratory toxicities is worth considering.

## AUTHOR CONTRIBUTIONS

**Lan‐Qing Huo:** Data curation (equal); methodology (equal); writing – original draft (lead). **Chu Chu:** Data curation (equal); formal analysis (equal); methodology (equal); writing – original draft (equal). **Xiaobo Jiang:** Data curation (equal); formal analysis (lead); methodology (equal). **Shiyang Zheng:** Project administration (equal); validation (equal); visualization (equal). **Pengxin Zhang:** Formal analysis (equal); methodology (equal); supervision (equal). **Rui Zhou:** Project administration (equal); software (equal); visualization (equal). **Nai‐Bin Chen:** Funding acquisition (equal); investigation (equal); resources (equal). **Jin‐Yu Guo:** Funding acquisition (equal); investigation (equal). **Bo Qiu:** Conceptualization (equal); writing – review and editing (equal). **Hui Liu:** Writing – review and editing (equal).

## CONFLICT OF INTEREST STATEMENT

The authors declared no conflict of interest.

## Supporting information


Table S1

## Data Availability

Research data are stored in an institutional repository and will be shared upon request to the corresponding author.
