# Supplementary material for: A pilot trial of consolidation bevacizumab after hypo‐fractionated concurrent chemoradiotherapy in patients with unresectable locally advanced non‐squamous non‐small‐cell lung cancer
Source: Cancer Med. 2023 Aug 3;12(17):17638–47. doi: 10.1002/cam4.6381 (PMC10523965; doi:10.1002/cam4.6381)
Supplement: Supplementary file 1 — Table S1 [file CAM4-12-17638-s001.docx]

*Supplementary Table 1.* Dose constraints of organs at risk

| Organs at risk | Dose constraints | |
| --- | --- | --- |
|  | The hypo-RT course  (40Gy/ 10fr) | The hypo-boost course  (24Gy/6fr or 28Gy/7fr) |
| Spinal cord | Dmax≤24Gy | Dmax≤12Gy |
| Lungs | V15≤20%, Dmean≤12Gy | V15≤10% |
| Heart | V30≤10% | V15≤10% |
| Esophagus | Dmax≤34Gy, V30<30% | Dmax≤24Gy, V20≤15% |

*Supplementary Table 2.* The dosimetry information

| Parameters | Study group (n=27) |
| --- | --- |
| Lungs V_20_ (%) |  |
| Median (Range) | 27.4 (10.3-36.5) |
| Mean dose of lungs (Gy) |  |
| Median (Range) | 16.5 (7.6-20.1) |
| Heart V_30_ (%) |  |
| Median (Range) | 8.8 (1.8-20.6) |
| Mean dose of heart (Gy) |  |
| Median (Range) | 11.2 (3.1-18.1) |
| Mean dose of esophagus (Gy) |  |
| Median (Range) | 26.7 (12.8-34.0) |
| Maximum dose of esophagus (Gy) |  |
| Median (Range) | 59.3 (21.0-68.8) |
| Maximum dose of spinal cord (Gy) |  |
| Median (Range) | 27.3 (10.4-39.7) |
